# Proposition d’algorithme décisionnel lors de discordances entre épreuves globulaire (Beth-Vincent) et sérique (Simonin) dans le groupage ABO au laboratoire d’immunologie du CHU de Bouaké (Côte d’Ivoire)

**DOI:** 10.48327/mtsi.v6i1.2026.814

**Published:** 2026-02-05

**Authors:** Lasme Roselle Charline MEMEL, Adjoumanvoule Honoré ADOU, Aya Ursule Aniela ASSI-SAHOUIN, Hebert Gautier KOYA

**Affiliations:** 1 Laboratoire d’immunologie du CHU de Bouaké, Côte d’Ivoire; 2 Laboratoire d’immunologie du CHU de Cocody, Abidjan, Côte d’Ivoire

**Keywords:** Groupage ABO, Discordance, Autoanticorps, Sécurité transfusionnelle, CHU, Bouaké, Côte d’Ivoire, Afrique subsaharienne, ABO Typing, Discrepancy, Autoantibodies, Transfusion Safety, University Hospital Center, Bouaké, Côte d’Ivoire, Sub-Saharan Africa

## Abstract

**Introduction:**

Le groupage sanguin ABO repose sur deux épreuves complémentaires : l’épreuve globulaire (Beth-Vincent) et l’épreuve sérique (Simonin-Michon). La concordance entre ces tests est essentielle pour la sécurité transfusionnelle. Les discordances persistantes, rares mais critiques, nécessitent une investigation approfondie.

**Méthodes et présentation des cas:**

Treize patients (6 à 73 ans) ont été explorés pour une discordance persistante ABO. Les tests parallèles Beth-Vincent et Simonin ont été réalisés, suivis, en cas de discordance, d’un triple lavage en solution saline des hématies. Les témoins auto, allo et AB ont été systématiquement effectués. Les contextes cliniques incluaient des traumatismes, des anémies, des bilans préopératoires et prénataux. Les antécédents étaient marqués, dans la majorité des cas, par une absence de transfusion antérieure. La majorité des cas présentait un témoin auto positif, évoquant des auto-anticorps, et/ou un témoin AB positif, suggérant une polyagglutinabilité. Les discordances concernaient principalement une opposition entre phénotype ABO direct et inverse (AB/O, A/O, B/O). Ces résultats montrent l’intérêt des témoins dans l’orientation diagnostique et la nécessité d’approches complémentaires.

**Conclusion:**

Cette série illustre l’algorithme décisionnel proposé en cas de discordances persistantes entre les épreuves globulaires et sériques lors du groupage sanguin ABO.

## Abstract (en)

**Introduction:**

ABO blood typing relies on two complementary tests: the red blood cell test (Beth-Vincent) and the serum test (Simonin-Michon). Concordance between these tests is essential for transfusion safety. Persistent discrepancies, though rare, are critical and require thorough investigation.

**Methods and case presentation:**

Thirteen patients (aged 6-73) were examined for persistent ABO discordance. We performed parallel Beth-Vincent and Simonin tests. In the event of discordance, we triple washed the red blood cells in saline solution. Auto-, allo-, and ABO controls were systematically performed. The clinical contexts included trauma, anemia, and preoperative and prenatal assessments. Most patients had no history of previous transfusion. Most cases presented a positive auto control, suggesting the presence of autoantibodies, and/or a positive AB control, indicating polyagglutinability. Discrepancies mainly concerned conflicts between direct and inverse ABO phenotypes (AB/O, A/O, and B/O). These results demonstrate the value of controls in guiding diagnosis and the need for complementary approaches.

**Conclusion:**

This series illustrates the decision-making algorithm proposed for cases with persistent discrepancies between red blood cell and serum tests during ABO blood typing.

**Figure:**
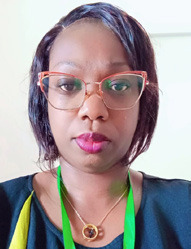

## Introduction

Le groupage sanguin ABO repose sur deux épreuves complémentaires : l’épreuve globulaire (Beth-Vincent), qui recherche les antigènes A et B à la surface des hématies, et l’épreuve sérique (Simonin Michon), qui permet de détecter les anticorps anti-A et anti-B présents dans le plasma [7]. La concordance entre ces épreuves est essentielle pour garantir la sécurité transfusionnelle et éviter les erreurs de groupage [15]. Une divergence entre les résultats de Beth-Vincent et de Simonin constitue avant tout un signal d’alerte, indiquant la nécessité d’une vérification approfondie et d’une investigation sérologique complémentaire, afin de prévenir toute incompatibilité ABO susceptible de provoquer des réactions hémolytiques aiguës, un choc, une insuffisance rénale ou un décès [3,12]. La règle dite « 4 × 2 » (deux techniques, deux techniciens, deux prélèvements, deux lots de réactifs) est recommandée pour minimiser les erreurs analytiques [11]. Toutefois, conformément à l’arrêté du 15 mai 2018 de la République française (JORF n°0116 du 23 mai 2018 ) fixant les conditions de réalisation des examens de biologie médicale d’immuno-hématologie érythrocytaire, et à l’instruction du 16 novembre 2021 relative à la réalisation de l’acte transfusionnel, il est précisé qu’en cas de technique manuelle, la détermination du phénotype érythrocytaire doit être effectuée à deux reprises sur le même échantillon biologique, par deux personnes habilitées différentes, chacune saisissant ses résultats de manière indépendante dans le système d’information du laboratoire [16]. Malgré ces conditions rigoureuses et les contrôles qualité mis en œuvre, certaines discordances peuvent persister entre les deux tests, nécessitant une investigation sérologique approfondie. Cette règle, issue des recommandations de bonnes pratiques transfusionnelles, contribue à réduire les risques d’erreurs pré-analytiques et analytiques [1,8,13]. La présente étude visait à rapporter 13 cas cliniques illustrant une discordance persistante entre les épreuves de Beth-Vincent et de Simonin, malgré le respect des procédures de lavage et des protocoles de contrôle qualité.

## Méthodes et présentation des cas

Les 13 cas ont fait l’objet d’un groupage ABO/RH selon la méthodologie suivante : tests parallèles Beth-Vincent et Simonin pour le groupage ABO et épreuve globulaire pour le groupage RH sur plaque d’opaline. Devant la discordance ABO, un lavage triple des hématies des patients a été réalisé à l’aide de solution saline isotonique, suivi après chaque lavage d’une centrifugation à 1 000 g pendant 5 minutes. Ce lavage à une température de 37 °C permet d’éliminer les protéines plasmatiques ou les anticorps libres qui pourraient interférer avec les réactifs d’hémagglutination, ce qui réduit les faux positifs [13,14]. Des contrôles qualité des sérums tests et hématies-tests ont été également effectués. La réalisation des témoins auto, allo et AB était systématique. Le témoin allo a consisté en l’ajout d’une goutte d’hématies d’un groupe O à 2 gouttes de plasma du patient, le témoin AB en deux gouttes de plasma AB plus une goutte de culot du patient et le témoin auto en une goutte de culot du patient mêlée à deux gouttes de son propre plasma.

**Figure 1:**
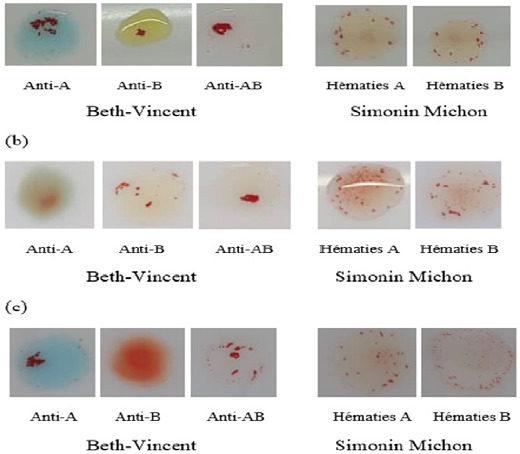
Discordances des deux épreuves (Beth-Vincent et Simonin Michon) observées au groupage ABO après lavage des hématies et dilution du plasma; (a) = AB/O, (b) = B/O et (c) = A/O

Les âges extrêmes étaient 6 ans et 73 ans (Tableau I). Les contextes cliniques étaient variés. Il s’agissait entre autres de traumatismes crâniens, d’anémie fébrile, de bilans préopératoires et prénataux. Les antécédents étaient marqués par une absence de transfusion sanguine dans la plupart des cas. En revanche, il y avait un cas de transfusion précoce et des terrains immunodéprimés au VIH. Plusieurs cas (ex. : cas 2, 3) présentaient un témoin auto et un témoin AB positifs, suggérant la présence d’autoanticorps. D’autres affichaient un témoin auto positif mais un témoin AB négatif, orientant vers une réactivité auto-spécifique. Le témoin AB positif dans certains cas pouvait évoquer une polyagglutinabilité. Les discordances concernaient principalement une opposition entre phénotype ABO direct et inverse (AB/O, A/O, B/O) (Fig. 1).

**Tableau I:** Informations relatives aux cas étudiés

Cas	Âge / Sexe	Contexte clinique	Antécédents	Résultats témoins (auto/allo/AB)	Observations
1	25 ans / M	Trauma crânien grave (urgence chirurgicale)	Absence de transfusion	Auto+ / Allo+ / AB+	Discordance AB/O persistante
2	6 ans/M	Syndrome anémique fébrile (pédiatrie)	Transfusion à J2 de vie	Auto+ / Allo- / AB+	Discordance A/O persistante
3	53 ans/M	Encéphalopathie (maladies infectieuses)	9 transfusions, VIH+	Auto+ / Allo+ / AB+	Discordance AB/O persistante
4	11 ans/M	BPO (chirurgie pédiatrique)	Absence de transfusion	Auto+ / Allo+ / AB-	Discordance AB/O persistante
5	55 ans / M	Dyspnée (urgences médicales)	VIH+ absence de transfusion	Auto+ / Allo+ / AB-	Discordance AB/O persistante
6	48 ans / M	BPO (externe)	Absence de transfusion	Auto+ / Allo+ / AB-	Discordance B/O persistante
7	28 ans/F	Bilan (gynécologie)	Absence de transfusion	Auto- / Allo+ / AB-	Discordance A/O persistante
8	48 ans/F	Anémie clinique (urgence médicale)	Absence de transfusion	Auto- / Allo+ / AB-	Discordance A / O persistante
9	17 ans / F	Anémie décompensée du post-partum (gynécologie)	Absence de transfusion	Auto+ / Allo+ / AB-	Discordance AB/O persistante
10	23 ans / F	BPO traumatisme crânien grave (gynécologie)	Absence de transfusion	Auto+ / Allo+ / AB-	Discordance AB/O persistante
11	46 ans/M	Bilan (anesthésie réanimation)	Absence de transfusion	Auto- / Allo+ / AB-	Discordance A/O persistante
12	73 ans/F	Bilan (oncologie)	Absence de transfusion	Auto- / Allo+ / AB-	Discordance AB/O persistante
13	57 ans / M	BPO (stomatologie)	Absence de transfusion	Auto+ / Allo+ / AB+	Discordance A/O persistante

BPO = bilan préopératoire Les résultats des témoins sont notés par « + » pour positif ou « – » pour négatif Le type de discordance est basé sur le groupe indiqué (Beth Vincent / Simonin) : AB vs O, A vs O, B vs O Le groupage RH était positif pour tous les cas étudiés

## Discussion

Les discordances persistantes entre les deux épreuves, malgré le lavage et les contrôles qualité, évoquaient plusieurs hypothèses. La présence d’autoanticorps (chauds ou froids) pouvait être en cause, en entraînant une agglutination non spécifique lorsque le témoin auto est positif [13,14]. Les autoanticorps chauds réagissent habituellement à 37 °C et peuvent agglutiner toutes les cellules, tandis que les autoanticorps froids (IgM) réagissent à basse température. Ces derniers sont le plus souvent polyclonaux dans les formes secondaires post-infectieuses, mais peuvent être monoclonaux lorsqu’ils sont associés à une hémopathie lymphoïde chronique [14].

Dans certains cas, comme chez les patients VIH positifs, l’immunodéficience peut être à l’origine de la discordance observée. Celle-ci résulte généralement d’une absence ou d’une faiblesse de réaction au test de Simonin, liée à un défaut de production d’anticorps, tandis que l’épreuve de Beth-Vincent reste le plus souvent normale, l’expression antigénique des hématies n’étant pas altérée [6]. Les effets liés à l’âge, notamment chez l’enfant (cas 2, 4) ou la personne plus âgée (cas 11), dont les taux d’anticorps peuvent être instables ou peu spécifiques avec une possibilité d’hypogammaglobulinémie, pourraient être aussi des causes de la persistance de la discordance ABO.

La polyagglutinabilité est une cause rare mais notable. Elle est un phénomène immunologique rare, dû à l’exposition anormale d’antigènes « cryptiques » sur les globules rouges, entraînant leur reconnaissance par des anticorps naturels présents dans la plupart des sérums humains [4]. Cette anomalie pourrait causer une discordance persistante entre les résultats globulaires et sériques, même après lavage, comme observé dans notre cohorte [4]. Ce phénomène doit être suspecté devant un témoin AB positif récurrent et confirmé par des tests enzymatiques ou moléculaires spécifiques [4].

Les sous-groupes antigéniques faibles (par exemple A2, A3) encore appelés phénotypes peu exprimés ou réactions atténuées ou absentes (par exemple discordance A / O), pourraient aussi entraîner des résultats discordants [2].

Ce travail met en avant l’importance d’une interprétation rigoureuse des résultats de groupage ABO, en intégrant systématiquement les témoins (auto, allo, AB) et en appliquant un algorithme décisionnel structuré [1,12,17]. En cas de discordance persistante, le recours à des techniques complémentaires, telles que le geltest ou le génotypage, peut être envisagé afin de garantir la fiabilité du résultat. Le génotypage est une approche moléculaire particulièrement utile pour élucider des situations complexes, en dehors des contextes d’urgence transfusionnelle où la rapidité d’exécution reste prioritaire. Bien qu’une plateforme de biologie moléculaire soit disponible à Bouaké, le génotypage ABO n’y est pas effectué en routine, en raison de l’absence de réactifs et de financement dédié à ce type d’examen. La gestion des discordances ABO repose exclusivement sur les méthodes sérologiques conventionnelles. En conséquence, malgré l’existence de l’infrastructure technique, le génotypage n’est pas accessible pour la routine diagnostique, et la gestion des discordances ABO repose exclusivement sur les méthodes sérologiques conventionnelles. Ainsi, tant que la discordance n’est pas résolue, la transfusion doit être sécurisée par l’utilisation de globules rouges de groupe O compatibles, afin d’éviter tout risque d’incompatibilité ABO [12,17]. Pour réduire les erreurs de groupage, il est essentiel d’adopter des protocoles standardisés et d’assurer une formation continue du personnel de laboratoire à l’échelle institutionnelle [1]. À plus long terme, établir un registre multicentrique des cas de discordance ABO pourrait améliorer les connaissances épidémiologiques, pour mieux cerner les causes sous-jacentes et optimiser les stratégies de prévention [18].

Au-delà de la simple description des 13 cas, cette étude met en évidence l’importance d’une démarche pratique pour le biologiste confronté à des discordances entre les épreuves globulaire (Beth-Vincent) et sérique (Simonin). Le lavage des hématies constitue une étape utile pour réduire l’interférence des anticorps naturels, mais il peut s’avérer insuffisant, notamment en présence d’agglutinines froides ou d’auto-anticorps. Dans ces situations, des approches complémentaires peuvent être envisagées, telles que l’adsorption sur plasma ou sérum pour neutraliser les auto-anticorps, ou la réalisation de l’épreuve de Simonin à 4 °C lorsque le test standard est affaibli [5].

## Recommandations pratiques et sécurité transfusionnelle

Il est important de rappeler que certaines discordances Beth-Vincent/Simonin peuvent persister malgré les investigations complémentaires. Dans ces cas, la priorité demeure la sécurité transfusionnelle. Il est recommandé de privilégier l’administration de concentrés de globules rouges de groupe O et de respecter strictement les procédures de compatibilité, afin de prévenir tout accident transfusionnel ABO [10]. L’intégration explicite de cette recommandation renforce le lien entre la démarche biologique et la prise en charge clinique, soulignant l’objectif ultime de ce type d’investigation : garantir la sécurité du patient.

## Conclusion

**Figure 2:**
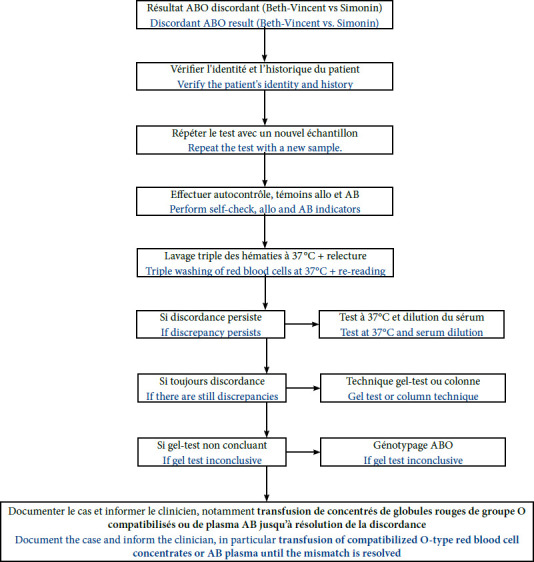
Algorithme décisionnel pour l’investigation d’un résultat ABO discordant entre les méthodes de Beth-Vincent et Simonin [12]. Le diagramme illustre les étapes successives, de la vérification de l’identité et de la répétition du test, aux techniques complémentaires (gel-test, génotypage ABO) et à la documentation du cas. L’étape finale notamment la documentation, la communication et la sécurité transfusionnelle sont essentielles. Ainsi pour la sécurité transfusionnelle, en cas de discordance ABO non résolue, il est recommandé d’utiliser uniquement des concentrés de globules rouges de groupe O compatibles et, si nécessaire, du plasma de type AB pour toute transfusion, jusqu’à ce que la discordance soit complètement élucidée.

Cette série de treize cas cliniques illustre la complexité du groupage ABO lorsque des discordances persistent malgré des conditions analytiques rigoureuses. Ces situations rappellent que la discordance entre les épreuves de Beth-Vincent et de Simonin doit avant tout être considérée comme un signal d’alerte, dont la reconnaissance immédiate constitue un maillon essentiel de la sécurité transfusionnelle. Les résultats soulignent également l’importance du recours systématique aux témoins (auto, allo, AB) et d’une démarche adaptative intégrant, selon le contexte, des techniques complémentaires. Enfin, nous proposons un algorithme décisionnel structuré (Fig. 2) pour renforcer la sécurité transfusionnelle face à ces situations rares mais critiques. Cet algorithme comporte une étape systématique d’autocontrôle, le recours à des techniques de gel et, au besoin, le génotypage ABO [9].

## Financement

Cette étude n’a reçu aucun financement externe.

## Contributions des auteurs et autrices

Lasme Roselle Charline MEMEL a défini le protocole de recherche, réalisé l’analyse statistique des données et rédigé la première version du manuscrit. Lasme Roselle Charline MEMEL et Hebert Gautier KOYA ont contribué à la collecte des données et à l’interprétation des résultats, Adjoumanvoule Honoré ADOU a assuré la supervision scientifique ainsi que la relecture du manuscrit. Aya Ursule Anela ASSI SAHOUIN a participé à la relecture de la version finale du manuscrit et à l’approbation finale.

Tous les auteurs et autrices ont approuvé la version finale.

## Déclaration de liens d’intérêts

Aucun lien d’intérêt n’a été déclaré.
