# Supplementary material for: A proposed decision-making algorithm for discrepancies between red blood cell (Beth-Vincent) and serum (Simonin) tests in ABO blood typing at the Immunology Laboratory of Bouaké University Hospital (Côte d’Ivoire)
Source: Med Trop Sante Int. 2026 Feb 5;6(1):mtsi.v6i1.2026.814. [Article in French] doi: 10.48327/mtsi.v6i1.2026.814 (PMC13216909; doi:10.48327/mtsi.v6i1.2026.814)
Supplement: Related texts

# Research article: A proposed decision-making algorithm for discrepancies between red blood cell (Beth-Vincent) and serum (Simonin) tests in ABO blood typing at the Immunology Laboratory of Bouaké University Hospital (Côte d’Ivoire)

## Introduction

ABO blood typing is based on two complementary tests: the globular test (Beth-Vincent), which detects A and B antigens on red blood cell surfaces, and the serum test (Simonin-Michon), which detects anti-A and anti-B antibodies in plasma [7]. Consistency between these tests is essential to ensure transfusion safety and avoid blood typing errors [15]. Discrepancies between Beth-Vincent and Simonin results primarily serve as warning signs, indicating the need for thorough verification and additional serological investigation. This is necessary to prevent ABO incompatibility, which could cause acute hemolytic reactions, shock, renal failure, or death [3,12].

The “4×2” rule (two techniques, two technicians, two samples, and two sets of reagents) is recommended to minimize analytical errors [11]. However, according to the French Republic’s May 15, 2018 decree (JORF No. 0116, May 23, 2018), which outlines the conditions for conducting medical biology tests for erythrocyte immunohematology, as well as the November 16, 2021 instruction regarding transfusion procedures, manual techniques require the erythrocyte phenotype determination to be performed twice on the same sample by two authorized individuals, with each entering their results independently into the laboratory information system [16].

Despite these rigorous conditions and quality controls, discrepancies may persist between the two tests, requiring further serological investigation. This rule, derived from recommendations for good transfusion practices, helps reduce the risk of pre-analytical and analytical errors [1,8,13]. The present study reports 13 clinical cases illustrating persistent discrepancies between the Beth-Vincent and Simonin tests despite compliance with washing procedures and quality control protocols.

## Methods and case presentation

Thirteen cases underwent ABO/Rh typing using the following methodology: parallel Beth-Vincent and Simonin tests for ABO typing, as well as a globular test for Rh typing, on an opaline plate. If ABO typing revealed discordance, the patients’ red blood cells were washed three times with an isotonic saline solution. After each wash, the cells were centrifuged at 1,000 g for five minutes. Washing at 37°C removes plasma proteins or free antibodies that could interfere with the hemagglutination reagents, thereby reducing false positives [13,14]. Quality controls of the test sera and red blood cells were also performed. Auto, allo, and AB controls were systematically performed. The allo control consisted of adding one drop of group O red blood cells to two drops of the patient’s plasma. The AB control consisted of two drops of AB plasma plus one drop of the patient’s pellet. The auto control consisted of one drop of the patient’s pellet mixed with two drops of their own plasma.

Patient ages ranged from 6 to 73 years (Table I). The clinical contexts varied. These contexts included head trauma, febrile anemia, and preoperative and prenatal assessments. In most cases, there was no history of blood transfusion. However, one case involved early transfusion and HIV-induced immunosuppression. Several cases (e.g., cases 2 and 3) had positive auto and AB controls, suggesting the presence of autoantibodies. Others had a positive auto control but a negative AB control, indicating auto-specific reactivity. A positive AB control in some cases could suggest polyagglutinability. Discrepancies mainly concerned a contrast between direct and inverse ABO phenotypes (AB/O, A/O, and B/O) (Fig. 1).

**Table I:** Information on the cases studied

Cas / Case	Âge / Sexe Age / Gendre	Contexte clinique / Clinical context	Antécédents / History	Résultats témoins (auto/allo/AB) / Control results (auto/allo/AB)	Observations / Observations
1	25 ans / M 25 years/ M	Trauma crânien grave (urgence chirurgicale) / Severe head trauma (surgical emergency)	Absence de transfusion / No transfusion	Auto+ / Allo+ / AB+ / Auto+ / Allo+ / AB+	Discordance AB/O persistante / Persistent AB/O discrepancy
2	6 ans/M 6 years/ M	Syndrome anémique fébrile (pédiatrie) / Febrile anemia syndrome (pediatrics)	Transfusion à J2 de vie / Transfusion on day 2 of life	Auto+ / Allo- / AB+ / Auto+ / Allo- / AB+	Discordance A/O persistante / Persistent A/O discrepancy
3	53 ans/M 53 years/ M	Encéphalopathie (maladies infectieuses) / Encephalopathy (infectious diseases)	9 transfusions, VIH+ / 9 transfusions, HIV+	Auto+ / Allo+ / AB+ / Auto+ / Allo+ / AB+	Discordance AB/O persistante / Persistent AB/O discrepancy
4	11 ans/M 11 years/ M	BPO (chirurgie pédiatrique) / POA (pediatric surgery)	Absence de transfusion / No transfusion	Auto+ / Allo+ / AB- / Auto+ / Allo+ / AB-	Discordance AB/O persistante / Persistent AB/O discrepancy
5	55 ans / M 55 years/ M	Dyspnée (urgences médicales) / Dyspnea (medical emergencies)	VIH+ absence de transfusion / VIH+ No transfusion	Auto+ / Allo+ / AB- / Auto+ / Allo+ / AB-	Discordance AB/O persistante / Persistent AB/O discrepancy
6	48 ans/M 48 years/ M	BPO (externe) / POA (external)	Absence de transfusion / No transfusion	Auto+ / Allo+ / AB- / Auto+ / Allo+ / AB-	Discordance B/O persistante / Persistent B/O discrepancy
7	28 ans/F 28 years/ F	Bilan (gynécologie) / Assessment (gynecology)	Absence de transfusion / No transfusion	Auto- / Allo+ / AB- / Auto- / Allo+ / AB-	Discordance A/O persistante / Persistent A/O discrepancy
8	48 ans/F 48 years/ F	Anémie clinique (urgence médicale) / Clinical anemia (medical emergency)	Absence de transfusion / No transfusion	Auto- / Allo+ / AB- / Auto- / Allo+ / AB-	Discordance A / O persistante / Persistent AB/O discrepancy
9	17 ans / F 17 years/ F	Anémie décompensée du post-partum (gynécologie) / Postpartum decompensated anemia (gynecology)	Absence de transfusion / No transfusion	Auto+ / Allo+ / AB- / Auto+ / Allo+ / AB-	Discordance AB/O persistante / Persistent AB/O discrepancy
10	23 ans / F 23 years/ F	BPO traumatisme crânien grave (gynécologie) / POA severe head trauma (gynecology)	Absence de transfusion / No transfusion	Auto+ / Allo+ / AB- / Auto+ / Allo+ / AB-	Discordance AB/O persistante / Persistent AB/O discrepancy
11	46 ans/M 46 years/ M	Bilan (anesthésie réanimation) / Assessment (anesthesia and intensive care)	Absence de transfusion / No transfusion	Auto- / Allo+ / AB- / Auto- / Allo+ / AB-	Discordance A/O persistante / Persistent A/O discrepancy
12	73 ans/F 73 years/ F	Bilan (oncologie) / Assessment (oncology)	Absence de transfusion / No transfusion	Auto- / Allo+ / AB- / Auto- / Allo+ / AB-	Discordance AB/O persistante / Persistent AB/O discrepancy
13	57 ans / M 57 years/ M	BPO (stomatologie) / POA (stomatology)	Absence de transfusion / No transfusion	Auto+ / Allo+ / AB+ / Auto+ / Allo+ / AB+	Discordance A/O persistante / Persistent A/O discrepancy

BPO = bilan préopératoire / POA = preoperative assessment Les résultats des témoins sont notés par « + » pour positif ou « – » pour négatif / Control results are marked with a “+” for positive or a “-” for negative Le type de discordance est basé sur le groupe indiqué (Beth Vincent / Simonin) : AB vs O, A vs O, B vs O / The type of discordance is based on the group indicated (Beth Vincent/Simonin): AB vs. O, A vs. O, B vs. O Le groupage RH était positif pour tous les cas étudiés / RH grouping was positive for all cases studied

**Figure 1:** Discrepancies between the two tests (Beth-Vincent and Simonin Michon) observed in ABO grouping after washing the red blood cells and diluting the plasma; (a) = AB/O, (b) = B/O, and (c) = A/O

## Discussion

Despite washing and quality controls, the persistent discrepancies between the two tests suggested several hypotheses. One possibility is the presence of autoantibodies (hot or cold), which can cause non-specific agglutination when the auto control is positive [13,14]. Hot autoantibodies typically react at 37°C and agglutinate all cells, whereas cold autoantibodies (IgM) react at low temperatures. The latter are most often polyclonal in secondary, post-infectious forms but can be monoclonal when associated with chronic lymphocytic leukemia [14].

In some cases, such as with HIV-positive patients, immunodeficiency may cause the observed discordance. This usually results in an absence or weak reaction to the Simonin test due to a defect in antibody production. However, the Beth-Vincent test usually remains normal because the antigenic expression of red blood cells is not altered [6]. Age-related effects, particularly in children (cases 2 and 4) and older adults (case 11), whose antibody levels may be unstable or nonspecific, with the possibility of hypogammaglobulinemia, could also cause persistent ABO incompatibility. Polyagglutinability is a rare but notable cause. It is an uncommon immunological phenomenon caused by the abnormal exposure of “cryptic” antigens on red blood cells. This leads to their recognition by natural antibodies present in most human sera [4]. This abnormality can cause persistent discordance between blood cell and serum results, even after washing, as observed in our cohort [4]. This phenomenon should be suspected when a recurrent ABO-positive control is present and confirmed by specific enzymatic or molecular tests [4].

Weak antigenic subgroups (e.g., A2 and A3), also known as attenuated or absent reactions (e.g., A/O discrepancy), could lead to discordant results [2]. This work emphasizes the importance of rigorously interpreting ABO grouping results by systematically integrating controls (auto, allo, and AB) and applying a structured decision algorithm [1,12,17]. In cases of persistent discordance, complementary techniques, such as gel testing or genotyping, may be employed to ensure the reliability of the results. Genotyping, a molecular approach, is particularly useful for elucidating complex situations outside of emergency transfusion contexts, where speed remains a priority. Although a molecular biology platform is available in Bouaké, ABO genotyping is not routinely performed due to a lack of reagents and funding dedicated to this type of testing. Consequently, the management of ABO incompatibilities relies exclusively on conventional serological methods. Consequently, despite the presence of the technical infrastructure, genotyping is unavailable for routine diagnosis, and the management of ABO incompatibilities depends entirely on conventional serological methods. Therefore, until the discrepancy is resolved, transfusions must use compatible type O red blood cells to avoid the risk of ABO incompatibility [12,17]. To reduce grouping errors, it is essential to adopt standardized protocols and ensure the ongoing training of laboratory staff at the institutional level [1]. In the long term, establishing a multicenter registry of ABO mismatch cases could improve epidemiological knowledge and help identify the underlying causes more effectively, thus optimizing prevention strategies [18].

Beyond describing the thirteen cases, this study emphasizes the importance of a practical approach for biologists when discrepancies arise between red blood cell (Beth-Vincent) and serum (Simonin) tests. Washing red blood cells reduces the interference of natural antibodies but may not be sufficient, especially when cold agglutinins or autoantibodies are present. In such cases, additional methods can be employed, such as adsorption on plasma or serum to neutralize autoantibodies or conducting the Simonin test at 4°C when the standard test is ineffective [5].

## Practical recommendations and transfusion safety

It is important to remember that Beth-Vincent/ Simonin discrepancies may persist despite additional investigations. In these cases, transfusion safety remains the priority. To prevent any ABO transfusion accidents, it is recommended to administer type O red blood cell concentrates and strictly follow compatibility procedures [10]. Including this recommendation explicitly strengthens the link between the biological approach and clinical management, emphasizing the ultimate goal of this type of investigation: ensuring patient safety.

## Conclusion

This series of thirteen clinical cases illustrates the complexity of ABO typing when discrepancies persist despite rigorous analytical conditions. These situations remind us that discrepancies between Beth-Vincent and Simonin tests are primarily a warning sign that must be recognized immediately to ensure transfusion safety. The results also underscore the importance of consistently employing controls (auto, allo, and AB) and adopting an adaptive approach that incorporates supplementary techniques based on the context. Finally, we propose a structured decision-making algorithm (Fig. 2) to enhance transfusion safety in these rare yet critical situations. The algorithm includes a systematic self-monitoring step, the use of freezing techniques, and ABO genotyping if necessary [9].

**Figure 2:** Decision algorithm for investigating an ABO mismatch between the Beth-Vincent and Simonin methods [12]. The diagram illustrates the successive steps, from identity verification and test repetition to complementary techniques (gel test, ABO genotyping) and case documentation. The final step, namely documentation, communication, and transfusion safety, is essential. Thus, for transfusion safety, in the event of unresolved ABO discordance, it is recommended to use only compatible O-type red blood cell concentrates and, if necessary, AB-type plasma for any transfusion until the discordance is completely resolved.

## Funding

This study received no external funding.

## Authors’ contribution

Lasme Roselle Charline MEMEL defined the research protocol, performed the statistical analysis of the data, and drafted the first version of the manuscript. Lasme Roselle Charline MEMEL and Hebert Gautier KOYA contributed to data collection and interpretation of the results, while Adjoumanvoule Honoré ADOU provided scientific supervision and proofread the manuscript. Aya Ursule Anela ASSI SAHOUIN participated in proofreading the final version of the manuscript and gave final approval.

All authors approved the final version.

## Conflicts of interest

The authors declare no conflicts of interest.
